# Radiochemotherapy Versus Surgery in Nonmetastatic Anorectal Neuroendocrine Carcinoma: A Multicenter Study by the Association des Gastro-Entérologues Oncologues

**DOI:** 10.1097/MD.0000000000001864

**Published:** 2015-10-23

**Authors:** Bertrand Brieau, Céline Lepère, Thomas Walter, Thierry Lecomte, Rosine Guimbaud, Sylvain Manfredi, David Tougeron, Françoise Desseigne, Nelson Lourenco, Pauline Afchain, Farid El Hajbi, Benoit Terris, Philippe Rougier, Romain Coriat

**Affiliations:** From the Department of Gastroenterology and Digestive Oncology, Cochin Teaching Hospital, Paris Descartes University, Paris, France (BB, RC); Department of Digestive Oncology, Georges Pompidou European Hospital, Paris Descartes University, Paris, France (CL, PR); Department of Gastroenterology, Edouard Herriot Hospital, Lyon, France (TW); Department of Gastroenterology, Tours Teaching Hospital, Tours Cedex 9, France (TL); Department of Medical Oncology, Claudius Regaud Institute, Toulouse, France (RG); Department of Gastroenterology, Rennes Teaching Hospital, Rennes, France (SM); Department of Gastroenterology, Poitiers Teaching Hospital, Poitiers, France (DT); Department of Medical Oncology, Leon Berard Hospital, Lyon, France (FD); Department of Gastroenterology, Saint Louis Hospital, Paris, France (NL); Department of Digestive Oncology, Saint Antoine Hospital, Paris, France (PA); Department of Medical Oncology, Oscar Lambret Hospital, Lille, France (FEH); and Department of Pathology, Cochin Teaching Hospital, Paris Descartes University, Paris, France (BT).

## Abstract

Neuroendocrine carcinomas (NEC) of the anus or the rectum are a rare disease, accounting for less than 1% of all digestive malignancies. Most are metastatic at diagnosis and treated with a platinum-based chemotherapy. No guidelines for localized tumors exist. The purpose of this study was to describe the characteristics of anorectal localized NEC, their management and their outcomes.

We retrospectively reviewed patients from 11 French centers with anorectal localized NEC. We compared 2 therapeutic managements: surgery (group A) versus chemotherapy with or without radiation (group B). Progression-free survival (PFS) and overall survival (OS) were estimated with the Kaplan–Meier method.

A total of 24 patients were identified with a median follow-up of 25 months (3–60 months). Median age was 63 years old and 17 had a rectal tumor (71%). Mean Ki-67 was 72% (range: 20–100), and 75% of the tumors had a high proliferative index (Ki-67 > 50%). Global PFS and OS were 13.1 and 44.1 months, respectively. Thirty-seven percent of patients were in group A and 63% in group B. There was no difference between group A and group B, whether in terms of PFS (13.0 months vs. 13.2 months, *P* = 0.75) or OS (49.1 months vs. 39.2 months, *P* = 0.42).

In patients with anorectal localized NEC, chemotherapy with or without radiation obtained a similar outcome as surgery and this conservative approach could be deemed a reasonable option.

## INTRODUCTION

Digestive neuroendocrine tumors are rare, accounting for less than 2% of all digestive malignancies in Europe and less than 1% in the United States.^1,2^ Among them, less than one-third are neuroendocrine carcinoma (NEC), with variable distribution according to countries, from 3.4% in Northern Europe to 30.3% in the United Kingdom.^3,4^ NEC are aggressive malignancies, mostly diagnosed in metastatic stage, defined by the 2010 World Health Organization (WHO) classification as poorly differentiated neuroendocrine neoplasm morphologically identical to small cell carcinomas of the lung and corresponding to grade 3 tumors (Ki-67 proliferative index >20% or >20 mitotic figures by 10 high-powered fields). NEC typically expresses neuroendocrine markers (eg, chromogranin, synaptophysin, neuron-specific enolase) explored by immunohistochemistry,^5^ and may secrete proteins such as chromogranin A whose levels are correlated with the stage of the disease and the tumor response.^6^

Gastrointestinal NEC are mainly metastatic, phenotypically and morphologically related to pulmonary high-grade neuroendocrine tumors, and managed likewise with a platinum-based chemotherapy with etoposide, leading to a median overall survival (OS) from 10 to 15 months.^7–9^ There is a paucity of data regarding the potential for local therapy to address nonmetastatic digestive NEC. However, as in small-cell lung cancer, surgical resection or radiochemotherapy might be considered.^10,11^ To date, there are no data confirming the overall benefit of local treatment in localized digestive NEC, and chemotherapy remains the mainstay therapy. Accordingly, the question of an aggressive approach for NEC of the anus and the rectum deserves an evaluation. We herein conducted a retrospective study of nonmetastatic NEC of the anus and the rectum to evaluate surgical and nonsurgical options.

## MATERIALS AND METHODS

### Patients

All consecutive patients with nonmetastatic NEC of the anus and rectum treated from January 2001 to December 2014 were eligible. The diagnosis of NEC was based on histological examination. Patients were included if they fulfilled the following criteria: a nonmetastatic NEC of the anus or the rectum and a therapeutic strategy including surgery, chemotherapy, and/or radiation. Patients were included in group A if they underwent surgery first and in group B if they received a chemotherapy regimen or a combined radiochemotherapy. Patients receiving a best supportive care approach without surgery or chemotherapy were excluded from the study.

Chest–abdominal–pelvic computed tomography scan was performed to ensure the absence of metastatic sites. Biopsies or surgical samples were checked by specialized pathologists in the framework of the RENATEN network (Réseau National de référence pour la prise en charge des Tumeurs Neuro-Endocrines malignes rares, sporadiques et héréditaires) confirming the diagnosis of NEC. The mitotic index was expressed as number of mitosis in 10 high-power fields (HPF) (2 mm^2^). The number of mitosis was determined in 40 fields taken in areas of highest mitotic density. The mitotic index corresponded to the sum of mitosis in the 10 richest fields.^5^ The KI67 index was determined using the Mib1 antibody and expressed as the percentage of labeled cells. The number of positive cells was determined from 500 to 2000 tumor cells in areas of higher density.

Patients with a well/moderate differentiation and/or a grade 1–2 proliferation index (Ki 67 < 20%) of the tumor, and/or metastatic disease were excluded. Patients with poorly differentiated adenocarcinoma or mixed adenoneuroendocrine carcinomas of the anus or rectum, whose tumors can expressed neuroendocrine markers similar to NEC,^12,13^ were also excluded.

This observational analysis was approved by the local ethical committee of Cochin hospital and thus meets the standards of the Declaration of Helsinki.

### Follow-Up

The follow-up after tumor resection or radiological response consisted in a clinical examination and a morphological imaging (chest–abdominal–pelvic CT scan), every 3 months for 2 years, then every 6 months for the following 3 years, or unless clinically indicated, according to RECIST version 1.0.^14^ The median follow-up was 25 months (3–60). Patients’ files were retrieved from the tumor registries of pathology departments and the medical information systems of each hospital and were reviewed for demographics, tumor characteristics, and first-line treatment (surgery, chemotherapy alone, or radiochemotherapy). Tumor evaluation was performed every 3 months.

### Statistical Analysis

The dates of progression and death were collected. The progression-free survival (PFS) was the period from the first day of therapy to disease progression. The OS was the period from the first day of the therapy to the death, induced or not by the tumor. Patients alive or lost during follow-up were censored.

Descriptive statistics [median, ranges, 95% confidence intervals (95% CIs)] were used to report patient baseline characteristics and treatment-induced adverse events. Comparisons between groups A and B were done using Fisher exact test, the χ^2^ test with Yates correction, or Wilcoxon test when appropriate.

All analyses assumed a bilateral type 1 error of 5%. Survival curves were estimated with the Kaplan–Meier method and compared with the log-rank test. Median follow-up was calculated with the reverse Kaplan–Meier method with GraphPad Prism program (GraphPad Software, La Jolla, CA).

## RESULTS

### Patients’ Characteristics

**Figure 1:**
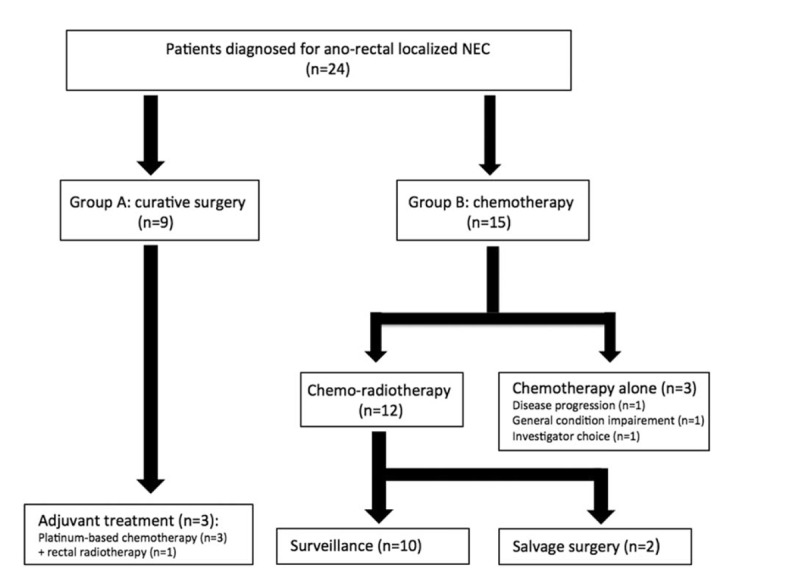
Flow chart. Distribution of treatments in 24 patients with nonmetastatic anorectal neuroendocrine carcinomas.

A total of 24 patients were included in the study. Mean Ki-67 was 72% (range: 20–100) and most of the tumors (75%) had a high Ki-67 proliferative index (>50%). Nine and 15 patients were included in the groups A and B, respectively (Fig. 1). The median age was 66.5 years (range 39–81) and 63 years (range 36–85) in groups A and B, respectively (Table 1). The global PFS and OS were 13.1 and 44.1 months, respectively.

**TABLE 1:**
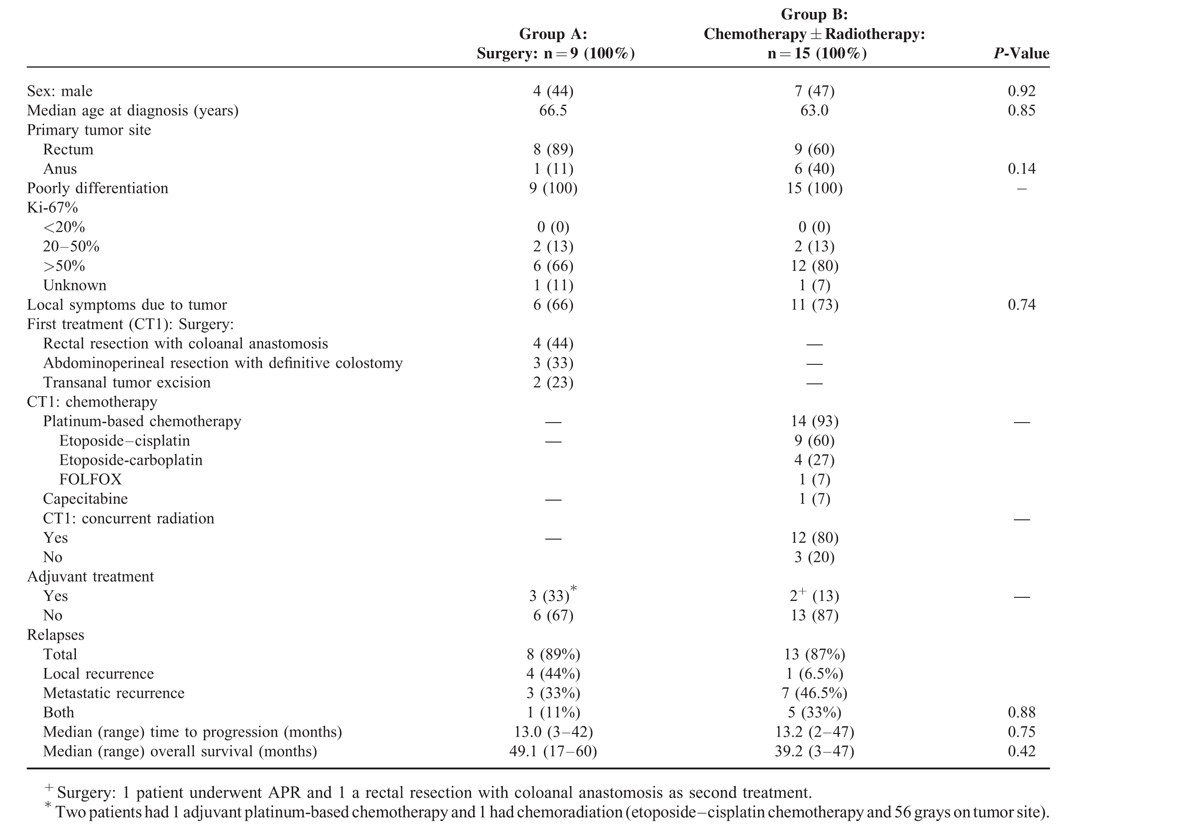
Patients’ Characteristics, Treatments, and Outcomes

### Treatment

In group A, surgery consisted in anterior rectal resection with coloanal anastomosis (4 patients), abdominoperineal resection with definitive colostomy (3 patients), and transanal tumor excision (2 patients). Two patients received adjuvant chemotherapy with etoposide–cisplatin (4 cycles) or 5-fluorouracil–cisplatin (4 cycles) regimen. One underwent adjuvant radiation (54 grays on tumor site) plus etoposide–cisplatin schedule (6 cycles).

In group B, 15 patients received chemotherapy with or without radiotherapy. Three of them (20%) were administered chemotherapy alone due to a poor general health status, a quick disease progression, or the investigators’ choice. The chemotherapy regimen was platinum-, etoposide-, and fluoropyrimidine based in 92%, 87%, and 13%, respectively, and the median number of cycles was 4 (range: 2–9). The main therapeutic choice was an etoposide plus cisplatin schedule (60%). Other chemotherapeutic options were etoposide plus carboplatin (27%), 5-fluorouracil plus oxaliplatin (6.5%), and capecitabin (6.5%). Twelve patients received radiation therapy. The radiotherapy was delivered from 40 to 50 grays and targeted the pelvic area to treat iliac nodes and the primary tumor. An additional boost of 15–20 grays on tumor site was performed in 10 cases. The mean dose of delivered radiation was 58 grays (range: 44–66). Two patients underwent surgical resection after the completion of the radiochemotherapy, the first one an anterior rectal resection with coloanal anastomosis and the other an abdominoperineal resection with definitive colostomy.

### Response to Treatment and Survival

**Figure 2:**
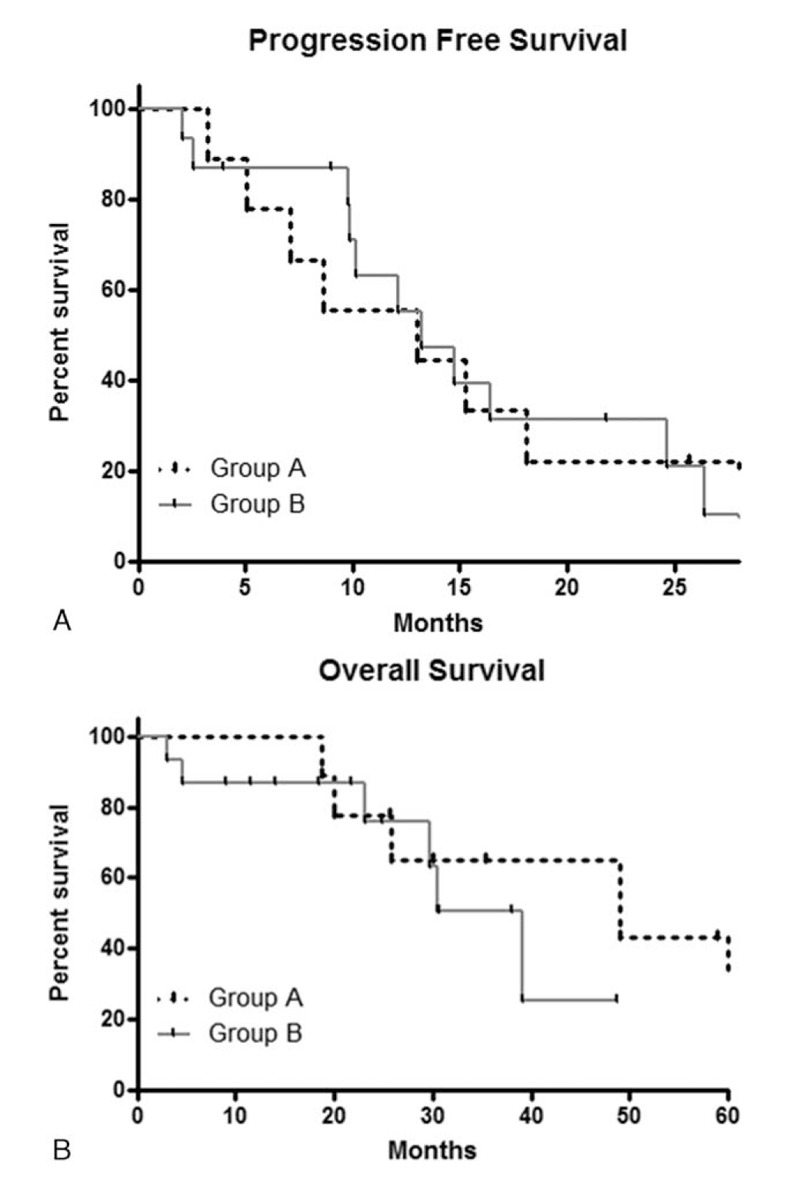
(A) Overall survival according to treatments. (B) Time-to-progression according to treatments.

In group A, 8 of the 9 operated patients had an R0 resection and 5 had a lymph node involvement. The disease recurred in 89% (n = 8) and relapse was local, metastatic, or both in 44%, 33%, and 11%, respectively. All patients experiencing a local relapse had undergone either a transanal excision or an anterior rectal resection with coloanal anastomosis. The median PFS was 13.1 months (range: 3–42) (Fig. 2A). The median OS was 49.1 months (range: 17–60).

In group B, patients experienced a complete response, a partial response, or a stable disease in 40%, 40%, and 13%, respectively. The disease control rate was 93%. One patient experienced at first evaluation a disease progression. The disease progressed in 87% (n = 13) and progression was local, metastatic, or both in 6.5%, 46.5%, and 33%, respectively. Two patients (13%) were disease free after a follow-up of 6 and 47 months. The median PFS and OS were 13.2 and 39.2 months, respectively.

No differences between groups A and B in PFS (*P* = 0.75; OR: 0.987 (0.58–1.39)), and OS (*P* = 0.42; OR: 1.253 (0.95–1.56)) were observed (Fig. 2A and B).

### Second- and Third-Line Treatments

In group A, all patients experiencing a local relapse underwent another surgical resection, and then relapsed with distant metastases. Treatment for metastatic patients consisted in chemotherapy, mainly platinum-based, from 1 to 4 lines of treatment.

In group B, most patients received chemotherapy after progression of the disease, from 1 to 3 lines of treatment. Two patients only had best supportive care without chemotherapy after the first progression.

## DISCUSSION

In the present study, we identified in nonmetastatic NEC of the anus or the rectum no benefit in PFS and OS in patients who underwent surgery rather than a conservative treatment with a loco-regional approach.

Localized anorectal NEC are very rare, and the different therapeutic managements of these lesions have not been compared. The guidelines of the main medical societies are also not supported by substantial scientific evidences and differ between themselves: the NANETS (North American Neuroendocrine Tumor Society) guidelines recommend to consider radiation and chemotherapy (cisplatin or carboplatin and etoposide for 4–6 cycles) while the ENETS (European Neuroendocrine Tumor Society) propose a surgical treatment in cases of localized digestive NEC.^15,16^

In our study, we identified a similar proportion of patients undergoing surgery or radiochemotherapy. Smith et al^17^ identified in a retrospective study including 126 patients with colonic and rectal NEC, a 20% incidence rate of patients with nonmetastatic anorectal NEC and identified the same proportion of surgical and nonsurgical approaches. In our study, we identified a disease control rate of 93% in line with Smith's study, which reported a similar response rate with chemoradiotherapy (92%), confirming the efficacy of a nonsurgical approach. In Smith's study, the median OS was 27 months in patients with nonmetastatic anorectal NEC, and surgery was not associated with a statistically significant difference compared with chemoradiotherapy. In our study, 88% of patients (n = 22) experienced a recurrence and there was no difference between surgery and chemoradiotherapy in terms of PFS (OR: 0.987 (0.58–1.39)) and OS (OR: 0.253 (0.95–1.56)). Eight of the 9 operated patients relapsed after a median time of 13 months. Two other patients underwent surgical resection following chemoradiation (CRT) for a tumor remnant, and experienced metastatic recurrences 5 and 17 months later. Thus, 91% of patients undergoing surgical resection in a curative intent relapsed. These results reinforced the lack of improvement of the PFS and the OS with a surgical management compared to the conservative option, whether in first or second intention.

In anal cancer, chemoradiotherapy has demonstrated its efficacy, obtaining a 5-year OS of 70% compared to 50% with surgery.^18,19^ Furthermore, the conservative management of anal canal cancer with CRT has avoided definitive colostomy for numerous patients—the standard surgical resection consisting in abdominoperineal resection with definitive colostomy (APR)—and is associated with a tolerable toxicity. Radiation therapy of anorectal tumor is frequently associated with gastrointestinal, genitourinary, and skin effects during the treatment, which spontaneously improve several weeks later. Late toxicities altering the quality of life such as radiation proctitis concern less than 20% of patients^20^ and are quite easily managed by medical treatment. Moreover, the toxicity should decrease in future with the generalization of intensity-modulated radiation therapy.^21^ Chemoradiotherapy has also demonstrated its efficacy in squamous cell carcinoma of the rectum.^22^

In low-rectum adenocarcinoma, surgical resection of the tumor remains the mainstay treatment but is associated with a high morbidity rate. Indeed, some patients undergo APR, a surgery whose postoperative complications are not rare, from 19% to 29% according to a recent study.^23^ Rectal cancer resection with a coloanal or a colorectal anastomosis exposes patients to functional sequelae such as anal incontinence. Additionally, definitive colostomy frequently affects the quality of life, social relationships, and causes psychological effects. The surgery of anorectal malignancies implies serious consequences in terms of perioperative complications, functional impairments, and quality of life, and should therefore be restricted to malignancies with a high probability of cure.

NEC are characterized by a high proclivity for metastatic dissemination even in patients with clinically localized tumors, and some retrospective studies have confirmed that surgery alone is rarely curative.^15,24,25^ These tumors are characterized by the poor differentiation and the high Ki-67 proliferative index whose rate is related with the tumor aggressiveness and probably with the potential of metastatic dissemination.^9^ In our series, 75% of patients had a Ki-67 > 50% and 67% a Ki-67 > 80%. Guidelines based on treatment paradigm for limited-stage small-cell cancer recommend chemotherapy (cisplatin or carboplatin and etoposide for 4–6 cycles) and radiation for loco-regional disease.^15^ Based on these data, resection of the primary tumor does not appear indicated. The only exception to this nonoperative approach may be for a symptomatic primary tumor; however, obstructed patients are rare in this setting and could be managed by endoscopic endoluminal stents or colostomy. Moreover, frequent symptoms such as pain or bleeding may be rapidly improved by chemotherapy or radiochemotherapy.

The best chemotherapy regimen is not defined in NEC. In our study, most patients had a platinum-based chemotherapy, which has showed its efficacy in metastatic NEC.^7^ The combination of etoposide and platinum salts, either cisplatin or carboplatin, seemed to be a reasonable option.

Some limitations should be noted concerning our study since its retrospective design and the relatively small number of patients represent its main flaws. However, patients of the 2 groups had comparable clinical characteristics with similar prognostics factors, such as the high Ki-67 proliferative index, allowing comparison of their therapeutic management.

In conclusion, nonmetastatic NEC of the anus or the rectum are aggressive tumors with similar poor prognosis to those of other localizations. Surgery does not improve PFS and OS compared to chemoradiotherapy alone. A schedule associating radiations at the tumor site and a doublet chemotherapy of 4 to 6 cycles by etoposide and carboplatin or cisplatin could be proposed awaiting prospective evaluation.

## References

[1] Yao JC Hassan M Phan A One hundred years after “carcinoid”: epidemiology of and prognostic factors for neuroendocrine tumors in 35,825 cases in the United States. *J Clin Oncol* 2008; 26:3063–3072. 18565894 10.1200/JCO.2007.15.4377

[2] Lepage C Bouvier AM Faivre J Endocrine tumours: epidemiology of malignant digestive neuroendocrine tumours. *Eur J Endocrinol* 2013; 168:R77–R83. 23349330 10.1530/EJE-12-0418

[3] Niederle MB Hackl M Kaserer K Gastroenteropancreatic neuroendocrine tumours: the current incidence and staging based on the WHO and European Neuroendocrine Tumour Society classification: an analysis based on prospectively collected parameters. *Endocr Relat Cancer* 2010; 17:909–918. 20702725 10.1677/ERC-10-0152

[4] Lepage C Ciccolallo L De Angelis R European disparities in malignant digestive endocrine tumours survival. *Int J Cancer* 2010; 126:2928–2934. 19569047 10.1002/ijc.24698

[5] Bosman FT Carneiro F Hruban R WHO Classification of Tumours of the Digestive System. 4th ed. 2010; Lyon, France: IARC Press, Chapter 8.

[6] Wang YH Yang QC Lin Y Chromogranin A as a marker for diagnosis, treatment, and survival in patients with gastroenteropancreatic neuroendocrine neoplasm. *Medicine* 2014; 93:e247. 25501094 10.1097/MD.0000000000000247 PMC4602794

[7] Moertel CG Kvols LK O’Connell MJ Treatment of neuroendocrine carcinomas with combined etoposide and cisplatin. Evidence of major therapeutic activity in the anaplastic variants of these neoplasms. *Cancer* 1991; 68:227–232. 1712661 10.1002/1097-0142(19910715)68:2<227::aid-cncr2820680202>3.0.co;2-i

[8] Mitry E Baudin E Ducreux M Treatment of poorly differentiated neuroendocrine tumours with etoposide and cisplatin. *Br J Cancer* 1999; 81:1351–1355. 10604732 10.1038/sj.bjc.6690325 PMC2362979

[9] Sorbye H Welin S Langer SW Predictive and prognostic factors for treatment and survival in 305 patients with advanced gastrointestinal neuroendocrine carcinoma (WHO G3): the NORDIC NEC study. *Ann Oncol* 2013; 24:152–160. 22967994 10.1093/annonc/mds276

[10] Sorensen M Pijls-Johannesma M Felip E Small-cell lung cancer: ESMO Clinical Practice Guidelines for diagnosis, treatment and follow-up. *Ann Oncol* 2010; 21 Suppl. 5:v120–v125. 20555060 10.1093/annonc/mdq172

[11] Schneider BJ Saxena A Downey RJ Surgery for early-stage small cell lung cancer. *J Natl Compr Canc Netw* 2011; 9:1132–1139. 21975913 10.6004/jnccn.2011.0094

[12] Gurzu S Serester O Jung I Possible neuroendocrine phenotype of poorly differentiated cell clusters in colorectal carcinoma, as a prognostic parameter. *Am J Surg Pathol* 2014; 38:143–144. 24335644 10.1097/PAS.0000000000000118

[13] Gurzu S Kadar Z Bara T Mixed adenoneuroendocrine carcinoma of gastrointestinal tract: report of two cases. *World J Gastroenterol* 2015; 21:1329–1333. 25632209 10.3748/wjg.v21.i4.1329 PMC4306180

[14] Therasse P Arbuck SG Eisenhauer EA New guidelines to evaluate the response to treatment in solid tumors. European Organization for Research and Treatment of Cancer, National Cancer Institute of the United States, National Cancer Institute of Canada. *J Natl Cancer Inst* 2000; 92:205–216. 10655437 10.1093/jnci/92.3.205

[15] Strosberg JR Coppola D Klimstra DS The NANETS consensus guidelines for the diagnosis and management of poorly differentiated (high-grade) extrapulmonary neuroendocrine carcinomas. *Pancreas* 2010; 39:799–800. 20664477 10.1097/MPA.0b013e3181ebb56f PMC3100733

[16] Ahlman H Nilsson O McNicol AM Poorly-differentiated endocrine carcinomas of midgut and hindgut origin. *Neuroendocrinology* 2008; 87:40–46. 17940332 10.1159/000109976

[17] Smith JD Reidy DL Goodman KA A retrospective review of 126 high-grade neuroendocrine carcinomas of the colon and rectum. *Ann Surg Oncol* 2014; 21:2956–2962. 24763982 10.1245/s10434-014-3725-3 PMC4521622

[18] James RD Glynne-Jones R Meadows HM Mitomycin or cisplatin chemoradiation with or without maintenance chemotherapy for treatment of squamous-cell carcinoma of the anus (ACT II): a randomised, phase 3, open-label, 2 × 2 factorial trial. *Lancet Oncol* 2013; 14:516–524. 23578724 10.1016/S1470-2045(13)70086-X

[19] Boman BM Moertel CG O’Connell MJ Carcinoma of the anal canal. A clinical and pathologic study of 188 cases. *Cancer* 1984; 54:114–125. 6326995 10.1002/1097-0142(19840701)54:1<114::aid-cncr2820540124>3.0.co;2-p

[20] Hayne D Vaizey CJ Boulos PB Anorectal injury following pelvic radiotherapy. *Br J Surg* 2001; 88:1037–1048. 11488787 10.1046/j.0007-1323.2001.01809.x

[21] Menkarios C Azria D Laliberte B Optimal organ-sparing intensity-modulated radiation therapy (IMRT) regimen for the treatment of locally advanced anal canal carcinoma: a comparison of conventional and IMRT plans. *Radiat Oncol* 2007; 2:41. 18005443 10.1186/1748-717X-2-41 PMC2204019

[22] Rasheed S Yap T Zia A Chemo-radiotherapy: an alternative to surgery for squamous cell carcinoma of the rectum—report of six patients and literature review. *Colorectal Dis* 2009; 11:191–197. 18462236 10.1111/j.1463-1318.2008.01560.x

[23] Schlussel AT Lustik MB Johnson EK A population-based comparison of open versus minimally invasive abdominoperineal resection. *Am J Surg* 2015; 209:815–823. 25766119 10.1016/j.amjsurg.2014.12.021

[24] Casas F Ferrer F Farrus B Primary small cell carcinoma of the esophagus: a review of the literature with emphasis on therapy and prognosis. *Cancer* 1997; 80:1366–1372. 9338459

[25] Brenner B Shah MA Gonen M Small-cell carcinoma of the gastrointestinal tract: a retrospective study of 64 cases. *Br J Cancer* 2004; 90:1720–1726. 15150595 10.1038/sj.bjc.6601758 PMC2409752

